# Smart Phages: Leveraging Artificial Intelligence to Tackle Prosthetic Joint Infections

**DOI:** 10.3390/antibiotics14090949

**Published:** 2025-09-19

**Authors:** Nicita Mehta, Andrew T. Nguyen, Edward K. Rodriguez, Jason Young

**Affiliations:** 1 Harvard Medical School, 25 Shattuck Street, Boston, MA 02115, USA; 2 Carl J. Shapiro Department of Orthopaedic Surgery, Beth Israel Deaconess Medical Center, Harvard Medical School, 75 Francis Street, Boston, MA 02115, USA; 3 Harvard Combined Orthopedic Residency Program, 55 Fruit Street, Boston, MA 02114, USA

**Keywords:** bacteriophage, phage therapy, machine learning, artificial intelligence, prosthetic joint infection

## Abstract

Traditional antibiotic therapy has encountered significant challenges for clinical treatment of infections for multiple reasons, including antimicrobial resistance (AMR) and poor efficacy against biofilms, demanding research into alternative therapeutic agents. Because of their unique antimicrobial mechanisms as well as their target specificity, diversity, exponential self-amplification, and anti-biofilm activity, combined with recent advances in genomics and synthetic biology, bacteriophages have attracted increased interest as potential alternatives or therapeutic adjuncts to antibiotics. However, obstacles such as phage-host specificity, bacterial resistance, and the selection of optimal phages, amongst other factors, impede clinical adoption of phage therapy. Here, machine learning (ML) and artificial intelligence (AI) tools have the opportunity to revolutionize phage therapy by enhancing scalability, efficiency and precision of these therapies. This article highlights potential key applications of ML/AI in the study, development and deployment of phage therapy.

## 1. Introduction

Antimicrobial resistance (AMR) is a significant source of morbidity and mortality globally. Overuse and misuse of antibiotics in multiple industries, decreased pharmaceutical innovation in antibiotic discovery and development, and intrinsic challenges of antibiotic resistance have contributed to rising rates of AMR, demanding research into alternative therapies for infection control and treatment. AMR has been an especially challenging complication in orthopedic surgery as arthroplasty-associated infection, or prosthetic joint infection (PJI) [1,2,3]. PJIs affect approximately 2% of primary arthroplasty patients and 3–5% of revision surgeries, leading to significant morbidity, prolonged hospitalizations, and revision surgeries. Traditional antibiotic therapy has struggled to treat PJIs for multiple reasons, including AMR and poor efficacy against biofilms on orthopedic implants, demanding research into alternative therapeutic agents [4,5].

Bacteriophages have attracted increasing attention for their specific targeting of pathogenic bacteria. Bacteriophages are viruses that specifically infect and, in the case of lytic bacteriophages, kill bacteria. Lytic phages replicate inside bacteria, co-opting bacterial translational machinery to produce phage-encoded lysins that disrupt bacterial cell walls, ultimately killing them. Because of this unique antimicrobial mechanism as well as their target specificity, diversity, exponential self-amplification, and anti-biofilm activity, combined with recent advances in genomics and synthetic biology, bacteriophages have attracted increased interest as potential alternatives for infection treatment [6,7].

Further, combining bacteriophages with antibiotics as adjuvant therapy is an emerging strategy to complement and, possibly, enhance the treatment of infections. Phages can target bacterial populations that are resistant to antibiotics, especially in biofilms, while antibiotics may further help suppress bacterial growth. This combination can produce synergistic effects, improving bacterial clearance and potentially reducing treatment duration. However, some phage-antibiotic pairs may interact antagonistically, diminishing efficacy. Even further, inappropriate use could accelerate resistance development or alter host microbial communities [8,9,10]. Careful selection and optimization of both phages and antibiotics are therefore critical for maximizing therapeutic benefit while minimizing risks; however, this challenge may be intractable with empiric experimental studies.

These phage-drug interactions in addition to other obstacles, such as phage-host specificity, bacterial resistance, and the selection of optimal phages, amongst other factors, impede clinical adoption of phage therapy [11,12,13,14,15]. Here, machine learning (ML) and artificial intelligence (AI) tools have the opportunity to revolutionize phage therapy by enhancing scalability, efficiency and precision of these therapies. Definitionally, AI broadly encompasses methods that perform tasks requiring human-like intelligence, such as reasoning, decision-making, or pattern recognition. More specifically, ML focuses on algorithms that integrate diverse inputs for a specified task. These methods can be powerful in managing large, complex, multi-dimensional datasets to identify subtle patterns and generate predictions or optimizations to support evidence-based decision making, image recognition, and natural language processing, amongst many other tasks [16].

This article highlights potential key applications of ML/AI in the study, development, and deployment of phage therapy for PJIs.

## 2. Predicting Phage-Host Interactions

Researchers and clinicians are increasingly interested in using bacteriophages to treat PJIs due to their highly specific targeting of bacteria. The molecular interactions responsible for this specificity depend on multiple complex mechanisms, including host receptor binding as well as possible defenses against viral infection. Molecular interactions are typically experimentally determined through methods such as co-immunoprecipitation or yeast two-hybrid screening. These approaches can be impractical for studying phage-host interactions due to the complex and highly specific binding dynamics between phages and diverse bacterial surface receptors as well as due to the number and diversity of combinations between bacterial and phage populations. Here, ML models, specifically supervised learning algorithms, such as random forests and neural networks, might be used to integrate protein structural, genomic, proteomic data to predict phage-host interactions. For example, more recently, Boeckaerts et al. employed a multilayer ML framework consisting of gradient boosting classifiers and protein language model embeddings to specifically predict strain-level phage-host interactions for *Klebsiella* species. Specifically, the authors leveraged protein features, such as receptor-binding protein sequences and bacterial surface characteristics, as numerical embeddings aggregated into multi-instance representations and inputted into gradient booster classifiers to predict potential phage-strain interactions. The authors demonstrated high accuracy (ROC AUC of 81.8%) in strain-level predictions to rank phage candidates for a bacterial strain, ultimately facilitating more focused laboratory investigation and enhancing precision in phage therapy development. Results were further validated both computationally and experimentally on high-risk *K. pneumoniae* isolates, outperforming traditional informed microbiologist-driven candidate selection strategies [17]. Similarly, Gaborieau et al. combined convolutional neural networks to extract features from bacterial and phage sequences with gradient-boosted decision trees to model phage infectivity in *Escherichia* strains and, ultimately, predict susceptibility with a focus on strain-specific interactions. The authors not only achieved high accuracy as benchmarked against empirically, experimentally determined phage-bacterial infection interactions, but also identified adsorption-related factors as critical determinants of phage infectivity [18]. These studies, amongst others demonstrate the ability of ML to accelerate and streamline identification of specifically effective phages for targeting of pathogenic bacteria to ultimately recommend tailored phage formulations for a targeted bacterium.

## 3. Development of Phage Libraries for Rapid Phage Selection

Complementarily, ML/AI can be used for the practical implementation of phage therapy in healthcare settings through the development of comprehensive phage libraries to streamline selection of therapeutic phages. Phage libraries are curated collections of characterized phages and include data on phage attributes such as host range, chemical stability, and lysis efficiency, amongst other factors. Clustering and natural language processing (NLP) algorithms might be used to group phages based on genetic homology or host specificity and extract data from the scientific literature and clinical reports, respectively. For example, Keith et al. constructed an input dataset of *E. coli* isolates tested against a library of phages to generate a matrix of isolate sensitivity to bacteriophage infection. This infection matrix, along with extracted genomic features from bacterial isolates, were used as input to a unique random forest classifiers to predict whether a given isolate would be infected by each phage. Even further, the authors synthesized these predictions to design optimized phage cocktails that were experimentally validated, ultimately demonstrating the ability for machine learning to rapidly recommend effective phage therapies [19]. Similarly, Yukgehnaish et al. employed an ensemble approach combining multiple classifiers to predict phage utility for therapeutic use by predicting lytic vs. temperate lifestyle and identifying genes associated with virulence and antibiotic resistance. Specifically, the authors curated a large dataset of publicly available phage genomes, annotated each genome, and extracted genomic sequence-based features that were converted into numerical representations as input for their ML pipeline. In this way, the authors developed a method to rapidly screen large phage libraries for safety and specific therapeutic efficacy [20]. Ultimately, ML/AI methods might provide a rapid, high-throughput computational filter to evaluate phage therapeutic potential, enable efficiency library organization, and quickly identification of therapeutic phages from decentralized biobanks.

## 4. Detection of Treatment Resistance

Evolutionarily, bacteria have developed mechanisms of resistance, such as CRISPR-Cas systems and membrane receptor mutations, to prevent phage infection. Identification and early detection of this resistance is critical in clinical settings to optimize treatment strategies and prevent ultimate treatment failure. In addition to long-read sequencing and hybrid assemblies [21], ML models, like time-series analyses and anomaly detection, might play a critical role in real-time, efficient monitoring of bacterial populations for signs of emerging resistance. Specifically, these methods integrate data analyses from sequential bacterial cultures or genomic sequencing to rapidly identify known or predicted mutations associated with resistance. For example, Feretzakis et al. evaluated the use of different machine learning architectures to predict AMR in multiple clinical isolates. Specifically, the authors used known resistance patterns and tested five models, including recurrent neural networks, logistic regression, decision trees, support vector machines, and random forests to classify strains as resistant or susceptible. While they focus on antibiotic resistance, this methodology might be generalized to monitor emerging resistance to phages by incorporating temporal phage susceptibility data [22]. By providing and integrating real-time data, ML methods might be used to ensure phage therapy efficacy throughout patients’ treatment course.

## 5. Personalization of Treatment

PJIs vary in complexity in multiple dimensions. They are often polymicrobial, heterogeneous bacterial populations that develop in patients with diverse biological factors, such as associated comorbidities and immune status, as well as infection characteristics, such as biofilm density and degree of bacterial diversity. This complexity not only requires thoughtful choice of phage but also consideration of combining multiple phages to target diverse strains, or phage cocktails. Designing effective phage cocktails can be challenging, as phages might interact antagonistically, and overuse can precipitate resistance. Previously, Pirnay et al. conducted a retrospective study of 100 personalized bacteriophage therapy cases, including PJIs, demonstrating clinical improvement using tailored phage selection methods as a personalized method for selecting phages for individual patients’ particular bacterial infections [23].

ML/AI methods might enable more personalized tailoring of phage therapy by integrating patient-specific data, bacterial characteristics, and phage attributes to predict treatment outcomes and optimize phage or phage-antibiotic combinations. ML models, such as support vector machines and reinforcement learning, might be able to not only predict treatment outcomes based on clinical and microbiological data, but also optimize phage cocktail formulations by modeling multi-dimensional interactions between bacteria, phages, antibiotics, and the host environment. For example, Kim et al. combined gradient-boosted decision trees and logistic regression models trained on genomic and phenotypic data of diverse *Pseudomonas aeruginosa* strains and resistance profiles to predict the efficacy of various phage-antibiotic combinations. The authors further tested effectiveness in an in vivo wound infection model of these phage-antibiotic cocktails, demonstrating the utility of ML methods to recommend specific, personalized regimens based on specific pathogens and resistance profiles [24]. By integrating multiple modalities of host, bacteria, and phage-specific data, ML/AI methods might enhance therapeutic precision and, ultimately, reduce the risk of treatment failure.

## 6. Challenges

Although ML/AI methods offer tools to support and accelerate the clinical implementation of phage therapy, there are a number of challenges that future research should consider in their use. First, generalizable and effective ML/AI methods rely on standardized, high-quality datasets to train robust models. However, due to the diversity of phage and bacterial strains, phage and bacterial genomic data are often inconsistent or incomplete. Second, ethical considerations, such as equitable access to support use of complex ML constructs, will need to be considered for global use, particularly in underserved clinical settings. Even further, ultimately integrating ML/AI methods into clinical workflows will require user-friendly interfaces.

## 7. Conclusions and Future Directions

Bacteriophages have garnered increasing interest as potential solutions to the increasing rates of AMR, particularly in the case of PJIs. ML and AI offer methods to accelerate the development, optimization, and clinical deployment of phages in healthcare settings by addressing key barriers in phage selection, cocktail design, resistance prediction, and treatment personalization. Here, we propose specific applications for the potential use of ML to accelerate the development and use of phage therapy for the targeted and effective treatment of PJIs and other complex antibiotic-resistant infections.

There are a number of other possible future directions that might be considered in the use of ML/AI in phage therapy development. This includes the development of synthetic phages to support more personalized therapy, as well as, from an infrastructure perspective, federated learning, in which ML models are trained across multi-institutional datasets that can accelerate the development of more generalizable phage libraries.

## Abbreviations

The following abbreviations are used in this manuscript:

PJI	Prosthetic Joint Infection
ML	Machine Learning
AI	Artificial Intelligence
NLP	Natural Language Processing
AMR	Antimicrobial Resistance
CRISPR	Clustered Regularly Interspaced Short Palindromic Repeats
